# The impact of therapeutic-dose induced intestinal enrofloxacin concentrations in healthy pigs on fecal *Escherichia coli* populations

**DOI:** 10.1186/s12917-020-02608-9

**Published:** 2020-10-08

**Authors:** Joren De Smet, Filip Boyen, Siska Croubels, Geertrui Rasschaert, Freddy Haesebrouck, Robin Temmerman, Sofie Rutjens, Patrick De Backer, Mathias Devreese

**Affiliations:** 1 Department of Pharmacology, Toxicology and Biochemistry, Faculty of Veterinary Medicine, Ghent University, Salisburylaan 133, 9820 Merelbeke, Belgium; 2 Department of Pathology, Bacteriology and Avian Diseases, Faculty of Veterinary Medicine, Ghent University, Salisburylaan 133, 9820 Merelbeke, Belgium; 3 Technology and Food Science Unit, Flanders Research Institute for Agriculture, Fisheries and Food, Brusselsesteenweg 370, 9090 Melle, Belgium

**Keywords:** *Escherichia coli*, Microbiota, Enrofloxacin, Antimicrobials, Antimicrobial resistance, Dose, Administration route

## Abstract

**Background:**

Knowledge of therapy-induced intestinal tract concentrations of antimicrobials allows for interpretation and prediction of antimicrobial resistance selection within the intestinal microbiota. This study describes the impact of three different doses of enrofloxacin (ENR) and two different administration routes on the intestinal concentration of ENR and on the fecal *Escherichia coli* populations in pigs. Enrofloxacin was administered on three consecutive days to four different treatment groups. The groups either received an oral bolus administration of ENR (conventional or half dose) or an intramuscular administration (conventional or double dose).

**Results:**

Quantitative analysis of fecal samples showed high ENR concentrations in all groups, ranging from 5.114 ± 1.272 μg/g up to 39.54 ± 10.43 μg/g at the end of the treatment period. In addition, analysis of the luminal intestinal content revealed an increase of ENR concentration from the proximal to the distal intestinal tract segments, with no significant effect of administration route. Fecal samples were also screened for resistance in *E. coli* isolates against ENR. Wild-type (MIC≤0.125 μg/mL) and non-wild-type (0.125 < MIC≤2 μg/mL) *E. coli* isolates were found at time 0 h. At the end of treatment (3 days) only non-wild-type isolates (MIC≥32 μg/mL) were found.

**Conclusions:**

In conclusion, the observed intestinal ENR concentrations in all groups showed to be both theoretically (based on pharmacokinetic and pharmacodynamic principles) and effectively (in vivo measurement) capable of significantly reducing the intestinal *E. coli* wild-type population.

## Background

Antimicrobial drugs are essential to treat clinical bacterial infectious diseases in both human and veterinary medicine. However, the extensive use of antimicrobials has led to an increase in antimicrobial resistance (AMR) [1]. A direct relation has been described between the use of antimicrobials and subsequent antimicrobial resistance [2–4]. Antimicrobial therapy does not only affect the targeted pathogen but also the commensal bacteria. Therefore, the gut microbiota is considered as a potential reservoir of resistance genes [5, 6]. The formation and selection of resistant strains in the gut commensal microbiota can facilitate the environmental spread of resistance genes and resistant bacteria [7]. This type of spread poses a significant risk for the animal to human resistance transfer and vice versa [8], pointing towards the need for a ‘One Health’ approach.

The currently marketed antimicrobials lack data on intestinal exposure. However, this exposure could have an effect on the formation or selection of resistant bacteria in the gut. Furthermore, limited information is available on the impact of the administration route and dose. Since parenteral administration does not require absorption from the gut, parenteral administration is generally deemed to have less influence on the gut microbiota compared with oral administration. For oral administration it has been demonstrated that incomplete absorption leads to gastro-intestinal drug residues which can affect the microbiota. However, not only incomplete absorption can cause intestinal exposure. Excretion of antimicrobials from blood to gut lumen can occur and is independent of administration route. Therefore, it is important to map the excretion mechanisms for specific molecules [9]. As previously reported, this excretion process is drug dependent [10]. In our previous study, sulfadiazine-trimethoprim was administered orally (PO) and intramuscularly (IM) to pigs and it was observed that sulfadiazine accumulated in distal gut segments and feces whilst trimethoprim displayed the exact opposite concentration pattern [10]. This accumulation was irrespective of the administration route. Peeters et al. [11] also observed a similar accumulation towards distal gastro-intestinal segments for tetracyclines after feeding cross-contamination levels of chlortetracycline and doxycycline to pigs.

Enrofloxacin (ENR) was selected as antimicrobial of interest to this study. It is a second-generation fluoroquinolone (FQ) and a structural analogue of ciprofloxacin (CIP), which is used in human medicine. ENR is administered to pigs for treatment of respiratory tract infections (e.g. *Pasteurella multocida* and *Actinobacillus pleuropneumoniae*) but it is also licensed (Federal Agency for Medicines and Health Products (FAMHP) of Belgium) to treat gastro-intestinal infections, e.g. caused by *E. coli*. The main purpose of this study was to elucidate the impact of the administration route on the intestinal concentrations of ENR in pigs. The other purpose was to assess the effect of these ENR intestinal concentrations on the coliform microbiota. Previously, it has been demonstrated that the FQ antimicrobials have a detrimental effect on the Gram-negative aerobic microbiota [12]. Römer et al. demonstrated that parenteral administration of ENR in piglets caused a considerable reduction of the susceptible intestinal *E. coli* population, in favor of resistant *E. coli* isolates [13]. Wiuff et al. also demonstrated a rapid development of resistance in coliforms in the gut of pigs after PO or IM administration of ENR [14]. However, no further pharmacodynamic (PD) and bacteriological investigation was executed. Therefore, it remains unclear whether the administration route and altered ENR dose could have an influence on resistance in the commensal microbiota and how this antimicrobial resistance is characterized.

In the current study, the effect of the administration route (PO or IM) on intestinal ENR concentrations was evaluated in pigs. Intestinal exposure can be related to incomplete gastro-intestinal uptake after oral administration (oral bioavailability) or to systemic intestinal excretion. Minimal dosing discrepancies were administered in order to simulate a possible in-field situation for the IM administrations. These dosing discrepancies were based on a publication by Callens et al. [15] after a survey of 50 different Belgian pig farms. This survey reported that oral antimicrobial treatment in pigs was often under-dosed, whilst IM treatment was mostly over-dosed. In Belgium, only IM administration of ENR is licensed for use in pigs (FAMHP). Oral administration of ENR was only evaluated to allow for a comparison with IM administration and to assess differences in intestinal exposure between oral and parental administration. From a One Health perspective, oral administration could also provide information when considering the pig as a model for human pharmacokinetics [16], since in human healthcare ciprofloxacin is also administered orally (tablets) and parenterally (intravenously). The main aim of this study was to determine the linearity of the ENR concentration in the different intestinal tract segments after oral or intramuscular administration of ENR at different doses. Secondarily, fecal samples were collected on different time points during treatment. The antimicrobial susceptibility of randomly collected *E. coli* isolates of these samples was examined. This experiment allowed for an evaluation of the impact of different ENR routes of administration (i.e. oral versus intramuscular) and doses on *E. coli* resistance selection in the porcine fecal microbiota.

## Results

### LC-MS/MS method validation

The results of the different validation parameters are given in supplementary Table B1 and B2 and fulfilled all criteria as described by the Veterinary International Conference of Harmonization (VICH, guideline 49 [17]) and the European Commission directives concerning the performance of analytical methods and interpretation [18]. The LC-MS/MS analytical method was based on a study using similar LC-MS/MS parameters for the determination of ENR in biological samples [19].

### Plasma, intestinal and fecal enrofloxacin concentrations

**Fig. 1:**
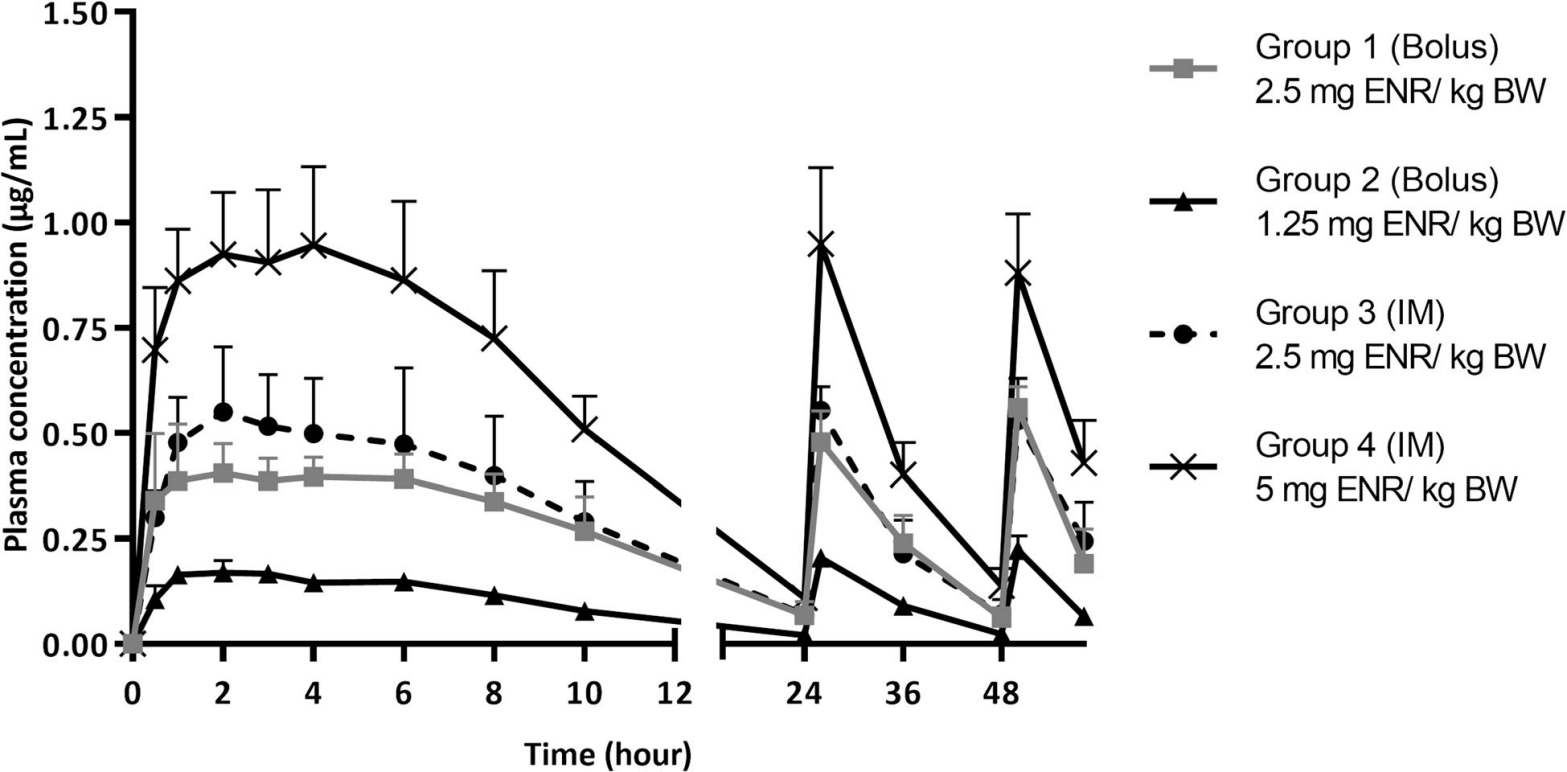
Plasma concentrations after the first administration of enrofloxacin (ENR) to pigs (*n* = 6 per group) and further during the three-day treatment period. Results are presented as mean + SD. With group 1(oral bolus) and 3 (IM): conventional dose: 2.5 mg/kg BW (1x daily); group 2 (oral bolus): half dose: 1.25 mg/kg BW (1x daily); group 4 (IM): double dose: 5 mg/kg BW (1x daily)

The plasma concentrations-time profiles of ENR for all treatment groups are displayed in Fig. 1. The PK parameters are given in Table 1 and were comparable to those in earlier studies of ENR in pigs [14, 20]. The AUC_0–24h_ values after one administration and AUC_0–58h_ values after a 3-day treatment period were compared between the different treatment groups (Table 1).

**Table 1:** Overview of the pharmacokinetic (PK) parameters in plasma

PK parameters	Group 1 PO 2.5 mg ENR/ kg BW	Group 2 PO 1.25 mg ENR/ kg BW	Group 3 IM 2.5 mg ENR/ kg BW	Group 4 IM 5 mg ENR/ kg BW
C_max_ (μg/mL)	0.45 ± 0.080^a^	0.18 ± 0.017^b^	0.57 ± 0.14^a^	1.02 ± 0.15^c^
T_max_ (h)	2.25 ± 1.48^a^	2.00 ± 0.63^a^	2.50 ± 0.84^a^	4.00 ± 2.10^a^
AUC_0–24 h_ (h*μg/mL)	5.93 ± 1.16^a^	2.02 ± 0.18^b^	6.88 ± 1.93^a^	12.26 ± 1.83^c^
AUC_0–58 h_ (h*μg/mL)	14.98 ± 2.13^a^	6.08 ± 1.40^b^	16.13 ± 3.87^a^	28.50 ± 3.65^c^
AUC_0–58 h_/D	8.96 10^− 4^ ± 4.63 10^− 4 a^	8.54 10^− 4^ ± 6.66 10^− 4 a^	8.59 10^− 4^ ± 3.81 10^− 4 a^	8.18 10^− 4^ ± 2.45 10^− 4 a^
Cp_ss_ (μg/mL)	0.25 ± 0.049^a^	0.092 ± 0.021^b^	0.29 ± 0.081^a^	0.51 ± 0.076^c^

Overview of the area under the curve from 0 to 24 h (AUC0–24 h), maximal plasma concentration of ENR (Cmax), and time of Cmax (Tmax) for groups 1, 2, 3 and 4 after the first administration (0–24 h). Also, the AUC0–58 h values (3 treatment days days), AUC0–58 h values normalized for dose (D) administered (AUC0–58 h/D) and the steady state plasma concentrations (Cpss) for groups 1, 2, 3 and 4 are given. A difference in superscripts a, b or c denotes a statistical significant difference between these groups. Statistics were exerted using a single-factor ANOVA with a post-hoc Tukey test (equality of variances checked), significance level 0.05. For AUC_0–58h_/D an independent-samples T test was used to compare the mean values between group 1–2 and 3–4

**Fig. 2:**
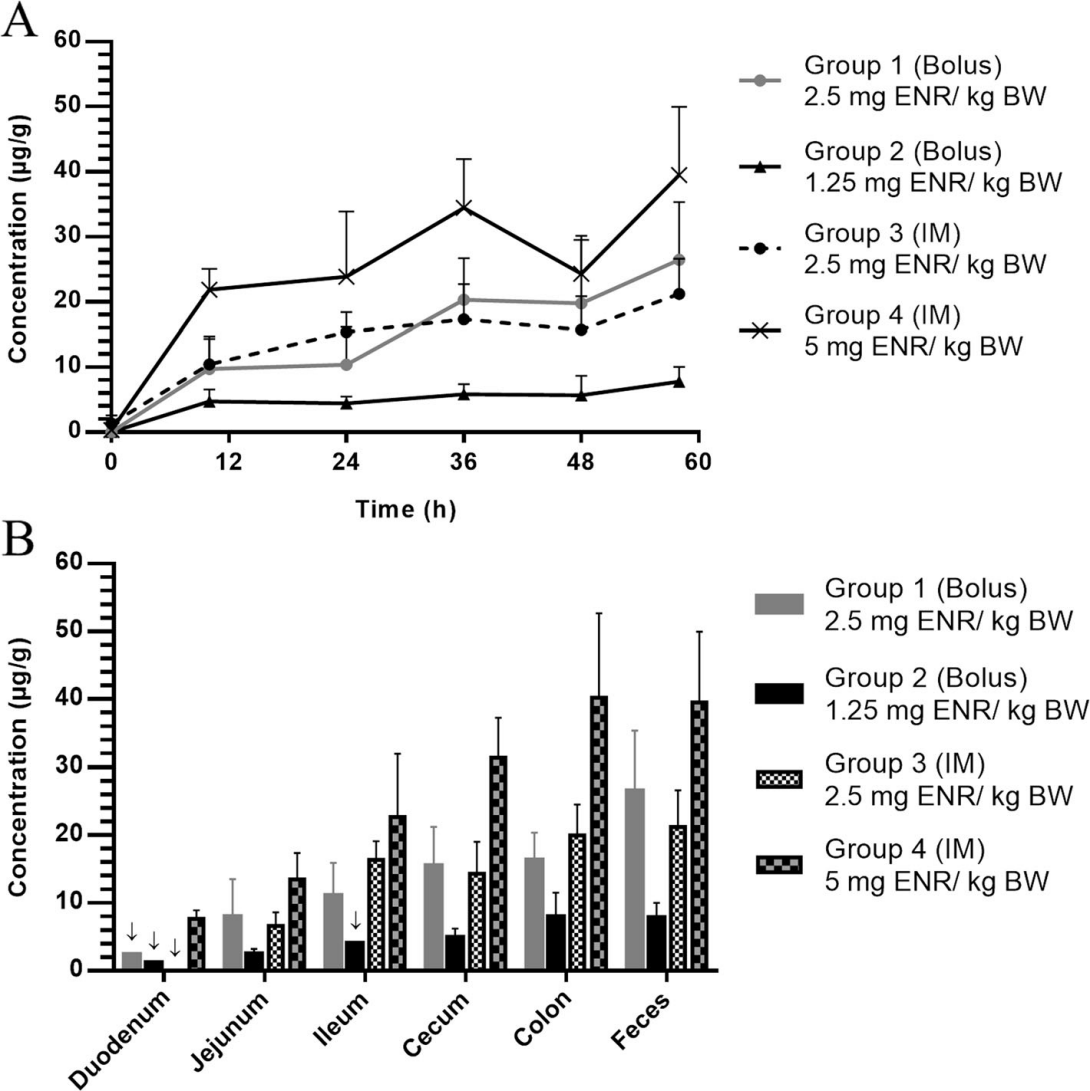
**a**: Enrofloxacin (ENR) concentrations in feces, collected during 3 days of treatment (sampling at 0, 10, 24, 34, 48 h and 58 h). With oral bolus administration in group 1 (*n* = 6): conventional: 2.5 mg/kg BW (1x daily); group 2 (*n* = 6): half dose: 1.25 mg/kg BW (1x daily); and IM administrations in group 3 (*n* = 6): conventional: 2.5 mg/kg BW (1x daily); group 4 (*n* = 6): double dose: 5 mg/kg BW (1x daily). **b**: ENR concentrations in the different gastro-intestinal segments: duodenum, jejunum, ileum, cecum, colon and feces. Sampling was performed 10 h after the final ENR administration (time point 58 h) in each treatment group. ↓ marking means no SD was presented because of the lack of sufficient data (either insufficient intestinal sample collection or values not quantifiable)

An overview of the average intestinal ENR concentration in six different gut segments is given in Fig. 2. The ENR concentration tends to increase towards the distal segments. A more than two-fold increase in concentration from jejunum to colon was observed in every treatment group. This observation was independent of the administration route (PO or IM).

In Fig. 2a, the average fecal concentrations of ENR for the different treatment groups are depicted. These were sampled twice daily during the 3-day treatment period. High fecal concentrations (ranging between 7.77 ± 2.24 μg/g and 39.54 ± 10.43 μg/g) were measured in every treatment group.

An overview of the calculated PK parameters based on the fecal concentrations is given in Table 2. No significant differences in AUC_0–58h feces_ values were found for group 1 and 3 (same dose, different administration route).

**Table 2:** Overview of the fecal pharmacokinetic (PK) parameters

PK parameters	Group 1 PO 2.5 mg ENR/ kg BW	Group 2 PO 1.25 mg ENR/ kg BW	Group 3 IM 2.5 mg ENR/ kg BW	Group 4 IM 5 mg ENR/ kg BW
C_max_ (μg/g)	17.75 ± 3.64^a^	5.11 ± 1.27^b^	15.00 ± 4.01^a^	24.14 ± 4.48^c^
C_max_/D	2.94 10^− 4^ ± 1.59 10^− 4 a^	2.6110^− 4^ ± 7.42 10^− 5 a^	3.67 10^−4^ ± 1.07 10^− 4 a^	2.97 10^− 4^ ± 1.17 10^− 4 a^
C_min_ (μg/g)	6.53 ± 4.96^a^	4.32 ± 1.51^a^	5.21 ± 4.97^a^	7.92 ± 4.01^a^
AUC_0–58hfeces_ (h*μg/g)	775.93 ± 217.80^a^	299.34 ± 55.55^b^	808.01 ± 122.33^a^	1469.61 ± 502.00^c^
C_ssfeces_ (μg/g)	12.94 ± 0.55^a^	4.51 ± 1.28^b^	10.64 ± 2.32^a^	19.53 ± 11.96^c^

Overview of the area under the curve from 0 to 58 h (AUC0–58 h) for fecal concentrations, maximal and minimal fecal concentrations (Cmax and Cmin) and average steady state fecal concentrations (Cpssfecal) for groups 1, 2, 3 and 4. Linearity was checked by normalizing Cmax for dose (D) administered (Cmax/D). A difference in superscripts a, b or c denotes a statistical significant difference between these groups. Statistics were exerted using a single-factor ANOVA with a post-hoc Tukey test (equal variances checked), significance level 0.05. For C_max_/D an independent-samples T test was used to compare the mean values between group 1–2 and 3–4

### Bacteriology

All selected isolates with score values of ≥2.300 after MALDI TOF-MS analysis were confirmed as *E. coli*. The majority (≥98%) of the investigated *E. coli* population at time point 0 h belonged to the wild-type (WT) population concerning ENR antimicrobial susceptibility. The magnitude of wild-type colony counts was in all treatment groups between 3-4 × 10^4^ CFU/g on MC agar. The median MIC was 0.023 μg/mL for group 1, 2 and 3 and 0.016 μg/mL for group 4. A minor (≤2%) non-wild type *E. coli* population was present in some animals at the start of the experiment (1 to 3 animals per group in the different treatment groups). The magnitude of the non-wild type colony counts was between 1-8 × 10^2^ CFU/g on MC agar + 0.125 μg/mL ENR. The median MIC was 1 μg/mL for group 1 and 2, 0.5 μg/mL for group 3 and 0.75 μg/mL for group 4. These isolates showed MIC values above the WT cut-off value, consistent with acquired ENR resistance mechanisms (0.5 μg/mL ≤ MIC≤2 μg/mL). At the end of the treatment (58 h), only a very low number of *E. coli* isolates was retrieved from the samples and the CFU count magnitude had dropped to ≤10^1^ CFU/g. Moreover, all of these isolates showed ENR MIC values of ≥32 μg/mL (32 μg/mL is the upper limit of the gradient test strip). An overview of the results obtained by sequencing the *gyr*A and *par*C genes for several wild-type (retrieved at 0 h), non-wild type (retrieved at 0 h) and all non-wild-type (retrieved at 58 h) isolates is given in Table 3. All non-wild type isolates collected at time point 0 h, carried silent mutations in *gyr*A (85GTT, 91CGT, 100TAC, 110TCC) and *par*C (91CAG). These isolates were also positive for the plasmid-mediated *qnr*S1 resistance gene [21], whereas the wild-type (retrieved at 0 h) and the non-wild type (retrieved at 58 h) isolates were negative for all investigated *qnr* genes.

**Table 3:** Results of the gene mutations of *gyr*A and *par*C for selected strains from the different treatment groups, the minimal inhibitory concentration (MIC) values are also provided for these isolates with wild-type cut-off of 0.125 μg/mL. The silent mutations, i.e. polymorphisms in codon sequence resulting in the same amino acid, in *gyr*A and *par*C are given, with the reference sequences indicated in grey. *Escherichia coli* K12 MG1655 was used for reference

Isolate	MIC (μg/mL)	***gyr***A mutations	***par***C mutations	*Silent mutations (gyrA/ parC) and additional resistance mechanisms*
***E. coli*** **K12** MG1655	NA	None	None	REFERENCE *gyr*A: 85GTC, 91CGC, 100TAT, 110TCT /*par*C: 91CAA
**ATCC 25922™**	0.064	None	None	
**Y01.V2 –** Group 1 – 0 h	0.064	None	None	
**Y08.V3** – Group 2 – 0 h	0.032	None	None	
**Y11.V2** – Group 3 – 0 h	0.016	None	None	
**Y12.V2** – Group 4 – 0 h	0.064	None	None	
**X02.V2 –** Group 1 – 0 h	2.00	None	None	*gyr*A: 85GTT, 91CGT, 100TAC, 110TCC/ *par*C: 91CAG and *qnr*S1 positive
**Y02.V2** – Group 2 – 0 h	4.00	None	None	*gyr*A: 85GTT, 91CGT, 100TAC, 110TCC/ *par*C: 91CAG and *qnr*S1 positive
**X06.V2 –** Group 2 – 0 h	4.00	None	None	*gyr*A: 85GTT, 91CGT, 100TAC, 110TCC/ *par*C: 91CAG and *qnr*S1 positive
**Y09.V2** – Group 3 – 0 h	2.00	None	None	*gyr*A: 85GTT, 91CGT, 100TAC, 110TCC/ *par*C: 91CAG and *qnr*S1 positive
**X12.V2** – Group 4 – 0 h	1.00	None	None	*gyr*A: 85GTT, 91CGT, 100TAC, 110TCC/ *par*C: 91CAG and *qnr*S1 positive
**X04.V2 –** Group 1 – 58 h	> 32.00	83Leu, 87Asn	80Ile	
**X07.V3 –** Group 2 – 58 h	> 32.00	None	None	
**X08.V4 –** Group 2 – 58 h	> 32.00	83Leu, 87Asn	80Ile	
**X09. V4 –** Group 2 – 58 h	> 32.00	83Leu, 87Asn	80Ile	
**X11.V6 –** Group 3 – 58 h	> 32.00	83Leu, 87Asn	80Ile	
**X13.V1 –** Group 4 – 58 h	> 32.00	83Leu, 87Asn	80Ile	
**X14.V1 –** Group 4 – 58 h	> 32.00	83Leu, 87Asn	80Ile	
**X15. V1** – Group 4 – 58 h	> 32.00	83Leu, 87Asn	80Ile	

*NA* Not available

**Fig. 3:**
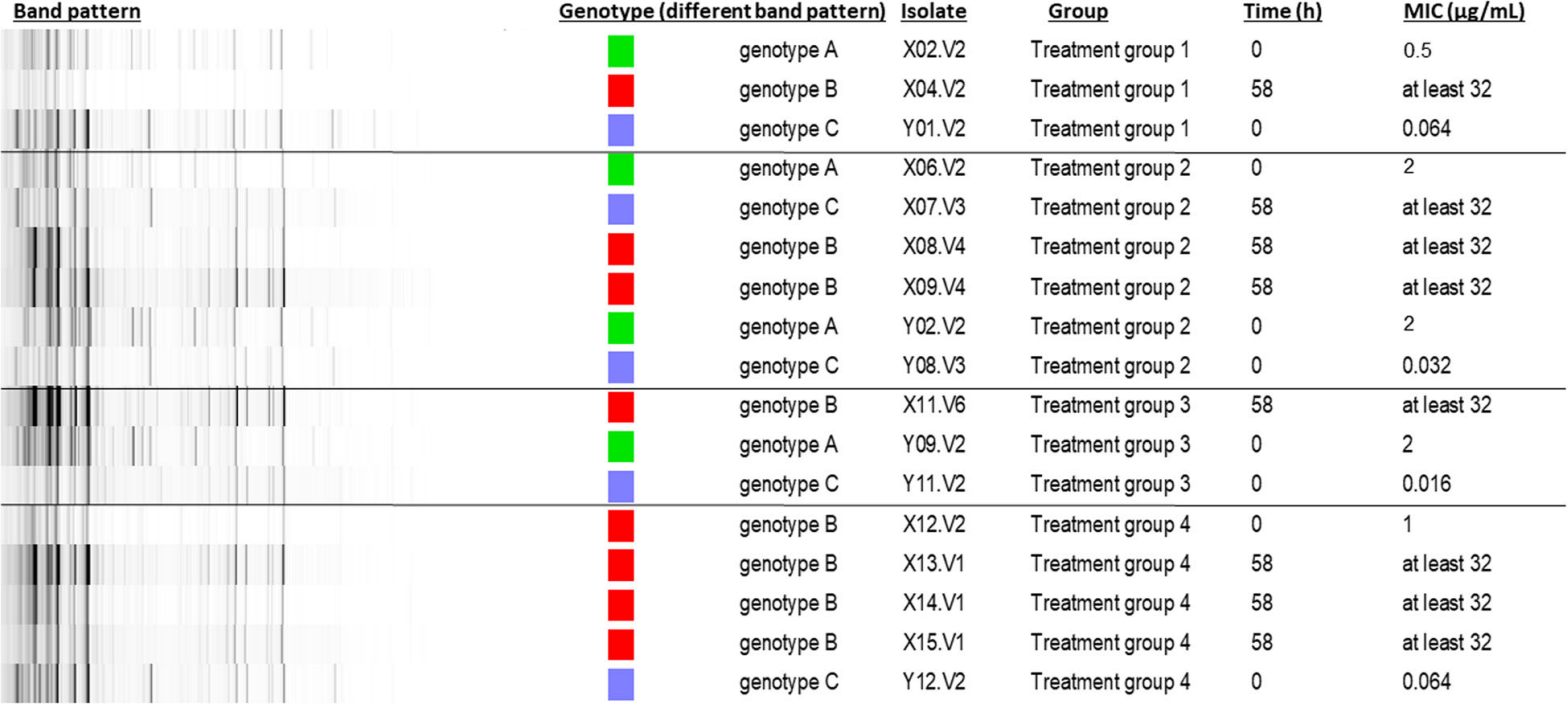
Repetitive element sequenced-based (rep)-PCR of 17 different retrieved *Escherichia coli* isolates (time point 0 h and 58 h), from the 4 different treatment groups and in total from 10 different pigs. The minimal inhibitory concentration (MIC in μg/mL) was determined for each isolate. Treatment group 1 and 2 were administered an oral bolus of either 2.5 or 1.25 mg enrofloxacin (ENR)/kg BW respectively (3 administrations), group 3 and 4 administered ENR intramuscular at 2.5 and 5 mg ENR/kg BW respectively (3 administrations). Different genotypic groups were observed. The different gel lines were re-grouped in order to sort the data per treatment group. This is also indicated in the figure by means of clear black lines

Next, all isolates were genotyped by (rep)-PCR as indicated in Fig. 3. The wild-type isolates (MIC≤0.125 μg/mL) that were tested, all belonged to genotype C. The non-wild type isolates (1 μg/mL ≤ MIC≤4 μg/mL) belonged to genotype A with one exception that belonged to genotype B. All non-wild type isolates with MIC≥32 μg /mL belonged to genotype B, with one exception belonging to genotype C. None of the non-wild type isolates with MIC≥32 μg /mL (both isolates belonging to genotype B as well as C) contained the gyrA or parC silent mutations or the qnrS1 resistance determinants as observed in the non-wild type isolates retrieved at 0 h.

## Discussion

### Plasma, intestinal and fecal enrofloxacin concentrations

There was no significant difference in AUC values between group 1 and 3. Both groups received the same dose (2.5 mg ENR/kg BW) but via a different administration route (PO and IM, respectively), indicating that the administration route had no significant influence on plasma concentrations of ENR. This is consistent with the report that the oral bioavailability of FQs in fasted pigs is complete [20]. Next, linearity of the dose was evaluated by normalizing the AUC values for the actual administered dose (D). The calculated AUC_0–58h_/D values were all in the same range within groups 1–2 (PO administration) and 3–4 (IM administration) (Table 1). Therefore, linear PK of ENR in plasma was assumed for both administration routes at the investigated dose range. After normalizing the measured fecal Cmax values for dose, linear PK was also confirmed for ENR in feces at the doses studied.

After conventional treatment (2.5 mg ENR/kg BW) either PO or IM, the average concentration of ENR was ≤10 μg ENR/g jejunal contents and increased to ≥20 μg ENR/g colonic contents with a maximum of 40.21 ± 12.49 μg/g. This increase in concentration towards the more distal segments indicates that the intestinal concentration is not related to an incomplete absorption process after PO administration. It should be noted that the intestinal concentrations that were measured during this study, represent a single time point (i.e. intestinal samples were collected at 10 h after the last administration of ENR) and do not display the dynamics of ENR movement within the GIT. Earlier work by Ferran et al. demonstrated the dynamics of marbofloxacin in the proximal intestinal segments, clearly demonstrating an alteration in concentrations of the molecule within the different GIT segments, in function of time (1.5, 2, 4, 6, 9, 12 and 24 h post-administration) [22]. However, the magnitude of these concentrations (5 up to 30 μg/g in the proximal segments) is comparable to the values reported in this study.

The observed high fecal concentrations of ENR are in line with the accumulating intestinal concentrations measured at the end of treatment (Fig. 2b). Furthermore, no significant differences (*p* > 0.05) in average concentrations of ENR were detected between the intestinal and fecal contents within the same treatment group. This indicates a similar concentration-related effect on the microbiota present in the distal gut and feces. It has to be mentioned that the measured concentrations of ENR do not differentiate between bound and unbound fraction in the feces or intestinal tract. The total amount of the molecule was measured in this study without making a distinction between antimicrobial active and inactive fraction. Nonetheless, this study shows the effect of the therapeutic strategy on the gastro-intestinal concentrations of ENR and the subsequent effect of these concentrations on *E. coli* isolates from the fecal microbiota. The goal was to compare the effect of different administration routes and doses within the same experiment. Given this relative comparison, the determination of the free antimicrobial fraction in the gut is less crucial.

High fecal concentrations of ENR in pigs have been reported before [23, 24]. However, renal excretion of ENR via glomerular filtration and active tubular secretion is still often reported as the main excretion mechanism [25]. The exact mechanisms of intestinal FQ secretion remain unclear. Several reports have indicated active intestinal secretion of FQ antimicrobials either through P-glycoprotein or cationic transporters [26–28]. Enterohepatic recirculation has also been described for ENR [29]. This can lead to an additional gastro-intestinal exposure. Lastly, ion trapping might also play a role in the increase of ENR in the distal gut segments because of the zwitterionic properties of ENR. At distal gut pH levels (≥6) a significant amount of the molecule (pKa 5.88 and 7.70) will be negatively charged [30]. This, in combination with the resorption of water in the distal parts of the gut, will attribute to the accumulation of ENR.

In conclusion, in this experimental setup, intestinal and fecal ENR concentrations are similar after oral and intramuscular administration of the same dose (2.5 mg ENR/ kg BW) during a three-day treatment period.

### PK/PD analysis

In general the most important PK/PD indices related to the efficacy of FQs are plasma C_max_/MIC≥10 [31] and AUC_0–24_/MIC≥125 h [32]. Extensive research has shown that these parameters correlate well with predicting the bacterial killing efficacy [31]. The fecal C_max_ values of ENR measured during the in vivo experiment (Table 2) indicate that at least in the distal parts of the intestines (colon, feces) and for all currently tested treatment protocols the *E. coli* isolates belonging to the ENR wild type population (MIC≤0.125 μg/mL) will be theoretically killed (C_max_/MIC≥10).

The C_max_ values from this study are based on the quantitative analysis of ENR in feces at 0, 10, 24, 34, 48 and 58 h during the three-day treatment period. These C_max_ values can be correlated to the specific MIC value of a bacterial isolate from the fecal microbiota. However, fecal and intestinal samples were not assessed frequently enough to generate an AUC from time point 0–24 h. Therefore, the AUC_0–24_/MIC parameter was not used in this study.

### Bacteriology

None of the non-wild type isolates (retrieved at 58 h, with MIC≥32 μg /mL) that belonged to genotype B contained the gyrA or parC silent mutations or the qnrS1 resistance determinants. Therefore, these isolates probably did not develop out of the non-wild type isolates (retrieved at 0 h). They may have been selected for from a parental strain, common to both the non-wild type genotype B isolates collected at 0 h and those collected at 58 h. Such a parental strain was not observed at the start of the experiment, but may have been missed, since not all wild type isolates could be genotyped. All of the non-wild type isolates (retrieved at 58 h) that belonged to genotype B carried mutations in *gyr*A (83Leu, 87Asn) and *par*C (80Ile). These mutations are associated with FQ resistance [33]. One non-wild type isolate (retrieved at 58 h) belonged to genotype C (X07.V3 from Table 3) and lacked mutations in *gyrA* and *parC.* This isolate was probably selected for out of the WT population since all investigated WT isolates observed at time point 0 h, belonged to genotype C. The mechanism of resistance for this isolate is not clear and requires further investigation, possibly indicating mutations in *gyrB* and/or *parE.* However, these mutations were not assessed during this study, as mutations in these genes are considered less prevalent for *E. coli* resistance against FQs [34]. Finally, there is an indication for clonal spread of a single high-resistant strain over the different treatment groups. Since, apart from X07.V3, all isolates belonged to the same genotype B.

The experimental setup applied in this study had some obvious shortcomings as it could not exclude (personnel-mediated) carry-over of genetic material between housing confinements of the treatment groups. In addition, (rep)-PCR, used to genotype the isolates, is known to lack sufficient reproducibility. In this context, whole genome sequencing could result in a higher accuracy and reliability. However, this technique is expensive and results in a high output, whereas (rep)-PCR is fast and inexpensive. In this study the isolates were genotyped by (rep)-PCR, since it is able to determine the genetic relatedness between the isolates. In future studies, where more detailed genotyping is needed, methods with higher discriminatory power should be applied. Another shortcoming in this study is the limited sensitivity of the applied detection method (plate enumeration). The fecal and intestinal samples were also frozen at − 80 °C without addition of a cryopreservant. This can reduce the number of counts for *E. coli*. However, in this study the plate counts of the different groups were compared. Therefore, absolute counts are less relevant. Nonetheless, a similar methodology for phenotypic analysis from fecal material is exerted by other related studies (albeit with different molecules). In these studies colony collection ranged from 3, 5 up to 20 colonies per plate respectively [35–37]. Since in this experiment there was no untreated control group, the accurate assessment of potential clonal spread was not possible. Taking into account these limitations, the presented results still indicate a significant impact of ENR on the fecal microbiota. Two studies using a similar methodology [13, 14] (i.e. plating of pig fecal material on a selective agar base to enumerate *E. coli* from the microbiota) demonstrated a similar susceptibility shift for *E. coli* from the fecal microbiota after treatment with ENR. In this context, *E. coli* is considered an important indicator bacterium, representing facultative anaerobic Gram-negative bacteria in the gut microbiota of animals and humans. It is the only bacterium of the fecal microbiota that was assessed in this study. However, in culture-based studies *E. coli* provides enough evidentiary value in terms of monitoring resistance. *E. coli* is also a bacterium of the microbiota in both humans and animals, and is abundant in the environment [38]. Therefore, it allows for a general estimation of resistance spread within this specific microorganism. In conclusion, limiting a bacteriological experiment to merely studying the effect in *E. coli* seems justifiable as these data actually provide indicative value. Nevertheless, the impact on the total microbiome can be different than solely based on *E. coli*.

At the end of the administration (58 h) only non-wild type isolates were recovered from the fecal samples. The presence of these non-wild type isolates is a remarkable finding and the cause of this observation is not clear. The fact that these isolates were not recovered at the beginning of the treatment could be related to their colonization site within the pig and because of the limited number of isolates that were fully characterized at time point 0 h. It is possible that minor resistant subpopulations were present in the mucus layers of the gut [39, 40] and were not found in the fecal samples. The composition of the intestinal microbiota can also differ from the one found in feces. Therefore, it cannot be excluded that the strain was present in the intestinal tract as a minor population, in one or more animals at the start of the experiment but was simply not detected until selective enrichment following ENR treatment. Nonetheless, de novo formation of these mutants cannot be excluded either. These bacteriological results are in alignment with the theoretical PK/PD predictions, for which a similar outcome was expected. It should be noted that these bacteriological determinations represent the situation of the fecal microbiota 10 h after the administration of the last dose of a three-day treatment period. It is expected that at this time point, the situation can be the most drastic in terms of resistance emergence in *E. coli* because of the sustained antimicrobial pressure during treatment. A post-treatment follow-up of the fecal microbiota after cessation of treatment was not exerted in this study. However, this could yield important results as a reversal of the observed resistance and eradication of fecal *E. coli* is likely to occur [41]. Nonetheless, in terms of this reversibility, several factors have to be taken into account such as fitness of the resistant mutants and dilution of the treated herd [42].

## Conclusions

This is the first study reporting relevant intestinal concentrations of ENR in pigs after both oral and parenteral administration. Intestinal ENR concentrations gradually increase from proximal towards distal gut segments. The highest concentrations were observed in colon and fecal samples. The observed intestinal ENR concentrations after both PO and IM administration demonstrated to be theoretically and effectively capable of reducing (i.e. below the limit of detection) the intestinal *E. coli* WT population. Further experiments are needed to determine whether dose or route of administration can influence either the magnitude or mechanisms of resistance selection.

## Methods

### Animal experiment

The animal experiment was approved by the Ethical Committee of the Faculties of Veterinary Medicine and Bioscience Engineering of Ghent University (case number EC 2015–16). The experimental setup and housing conditions were in complete accordance with the Belgian law, as stated in the Royal Decree of the 29th of May 2013 “KB on the protection of experimental animals”. Twenty four pigs (Belgian Landrace, 10 weeks old, mixed sex and not exposed to previous antimicrobial treatment) were obtained from Flanders Research Institute for Agriculture, Fisheries and Food (Melle, Belgium). The pigs were group-housed in the same stable but within different, fully separated confinements (*n* = 3 per confinement) on 50/50 concrete floor/grids and had ad libitum access to food (Aveve NV, Melle, Belgium) and water during the entire study. An acclimatization period of 5 days was respected before the start of the experiment. The animals had never been treated with FQ antimicrobials before the start of the experiment. Each group consisted of six animals, with the group as experimental unit and plasma/ faecal concentration of ENR as primary parameter. The number of animals was based on previously obtained data.

Baytril® 10% oral solution was used (Bayer SA-NV, Diegem, Belgium) for the oral administrations in group 1 (average bodyweight (BW): 16.17 ± 3.19 kg) and 2 (average BW: 15.83 ± 1.17 kg). Oral administration was done via oral gavage for 3 consecutive days, with a 24 h interval per administration. Group 1 was administered a dose of 2.5 mg ENR/kg BW in accordance with the leaflet, and group 2 was administered half the recommended dose, namely 1.25 mg ENR/kg BW. The animals in group 3 (average BW: 16.83 ± 2.99 kg) and 4 (average BW: 16.33 ± 1.63 kg) received the dose of ENR via IM administration with Baytril® 100 mg/mL (Bayer SA-NV, Diegem, Belgium), which was also administered once daily with a 24 h interval for 3 consecutive days, as described by the manufacturer. Group 3 was administered an injection of 2.5 mg ENR/kg BW in accordance with the leaflet, and group 4 was administered a double dose of 5 mg ENR/kg BW. An overview of the experimental setup is given in Figure A (supplementary files). During the first administration-day, blood samples (± 1 mL) were collected from the jugular vein in heparin-containing vacuum tubes (Vacutest Kima, Arzergrande, Italy) at time points: 0, 0.5, 1, 2, 3, 4, 6, 8, 10 and 24 h after administration. Furthermore blood was collected daily on the remaining treatment days; i) pre-administration, ii) at expected time of maximal plasma concentration (2 h) and iii) at expected trough concentrations (10 h post-administration). Fecal samples (± 2 g) were collected daily; i) pre-administration and ii) 10 h post-administration in sterile plastic cups, after rectal stimulation. After 3 days of treatment, the animals in each treatment group were euthanized 10 h after the last administration (i.e. 58 h for IM groups and 106 h for oral groups after start of dosing), with induction of anesthesia (0.3 mg/kg BW xylazine (Xyl-M®, V.M.D. Vet, Arendonk, Belgium) and 15 mg/kg BW tiletamine-zolazepam (Zoletil 100®, Virbac, Barneveld, the Netherlands)) followed by intra-cardiac injection of sodium pentobarbital 20% (Kela Veterinaria, Sint-Niklaas, Belgium). Next, intestinal content (± 2 g whenever possible) was collected from different gut segments (duodenum, mid-jejunum, ileum, cecum, mid-colon and rectum). Blood samples were centrifuged (2851 x g, 10 min, 4 °C) and plasma was separated and stored at ≤ − 15 °C within 2 h after collection. Fecal samples and intestinal content were stored at ≤ − 80 °C within 2 h after collection.

### LC-MS/MS analysis of ENR in plasma and intestinal content

#### Chemicals and reagents

All solvents used were of analytical grade; acetonitrile (ACN), methanol (MeOH), water (H_2_O) from Fisher Scientific (Erembodegem, Belgium), glacial acetic acid and ethyl acetate from VWR (Leuven, Belgium). Standards of ENR and internal standard (IS) ENR-d5 were purchased from Sigma-Aldrich (Diegem, Belgium) and prepared in a H_2_O/MeOH solution (50/50 v/v). These stock solutions of 1 mg/mL were stored airtight and protected from light at ≤ − 15 °C for a maximal period of 60 days. Phosphate-buffered saline (PBS) was purchased from Sigma-Aldrich (Diegem, Belgium).

#### Sample preparation

The sample preparation for plasma, fecal and intestinal samples was very similar. For all fecal and intestinal samples, one gram of sample was weighed for further quantitative analysis. These samples were diluted 10-fold (weight based) in PBS. The fecal and intestinal samples were spiked with 25.0 μL of the IS solution (40 μg/mL ENR-d5 in 50/50 (v/v) H_2_O/MeOH). After liquid-liquid extraction with ethyl acetate and a horizontal shaker (10 min), all samples were evaporated to dryness at 40 ± 2 °C with nitrogen. The extract was reconstituted using 500.0 μL of a H_2_O/ACN (80/20 v/v) mixture. Finally, the samples were transferred to a glass vial after filtering through 0.45-μm nylon filters (Merck Millipore, Overijse, Belgium). An aliquot of 10.0 μL was injected onto the liquid chromatography-tandem mass spectrometry (LC-MS/MS) instrument. For the plasma samples, 250 μL of plasma was spiked with 12.5 μL of the IS solution (10 μg/mL ENR-d5 in 50/50 (v/v) H_2_O/MeOH), extracted with ethyl acetate (shaken for 10 min) and evaporated to dryness under nitrogen flow 40 ± 2 °C. The samples were reconstituted with 250.0 μL of a H_2_O/ACN (80/20 v/v) mixture. Again, an aliquot of 10.0 μL was injected onto the LC-MS/MS instrument.

#### LC-MS/MS analysis

For liquid chromatography a Zorbax Eclipse Plus column (Reversed Phase C18, 100 mm × 30 mm i.d., dp: 3.5 μm) in combination with a guard column (13 mm × 3 mm i.d., dp: 3.5 μm) was used (Agilent Technologies, Diegem, Belgium). Mobile phases and gradient elution for chromatographic separation are given in supplementary Table A1. The LC effluent was coupled to a Thermo Fisher Scientific TSQ® Quantum Ultra (Breda, The Netherlands) triple quadrupole mass spectrometer with ion source heated electrospray ionization (ESI) operating in positive ionization mode. Acquisition was performed in the selected reaction monitoring (SRM) mode. For ENR and IS, the following transitions were followed (*quantification ion): ENR: *m/z* 360.0 > 316.07, 244.74* and ENR-d5: *m/z* 365.0 > 321.11, 244.81*. Further details of the instrumentation parameters are given in supplementary Table A2. The methods used for quantification of ENR in this study were validated using matrix-matched calibrator and quality control samples. These were based on blank plasma and fecal samples originating from untreated pigs. The method validation was based on an in-house developed validation protocol as described by De Baere et al. [43].

### Isolation, quantification and characterization of *E.coli* strains from faeces

For bacteriological investigations, fecal samples from each animal were examined. The samples were obtained at the beginning of the experiment just before treatment (0 h) and 10 h after the last administration of ENR (58 h). These fecal samples (1 g weighed) were thawed from ≤ − 80 °C and diluted 10-fold (weight-based) in sterile PBS. Next, 40.0 μL of this dilution was plated onto i) MacConkey (MC) agar n° 3 (Oxoid NV, Erembodegem, Belgium) and ii) MC agar supplemented with 0.125 μg/mL ENR (EUCAST epidemiological cut-off (ECOFF)). Spiral plating was performed with an Eddy Jet spiral plater (IUL S.A., Barcelona, Spain) to enumerate the colonies. Subsequently, the agar plates were aerobically incubated at 35 ± 2 °C for 20–24 h. Total plate count was measured by manual count on both sets of plates. Plate counts were performed on the dilutions that resulted in a colony density of 20–300 colonies per plate [44]. Only regular-shaped, large lactose positive (pink) colonies were provisionally identified as *E. coli* and were counted. Up to 5 colonies (when available) per plate were purified and identified by means of Matrix-Assisted Laser Desorption Ionization-Time-of-Flight Mass Spectrometry (MALDI-TOF MS) analysis. Briefly, a random purified colony was picked from the agar plate and spread out on a polished steel target plate. These spots were covered with 1.0 μL of α-cyano-4-hydroxycinnamic acid (HCCA) matrix, according to the manufacturer’s guidelines. The spectra were obtained and analyzed with the MBT Compass software version 4.1 (Bruker Daltonik), which included a database of 6120 mean spectra projections (MSP). The analysis was repeated when score values < 2.000 were obtained. Genotyping of *E. coli* isolates was performed using repetitive element sequenced-based (rep)-PCR, as described by Peeters et al. [45].

### MIC determination

Purified and identified *E. coli* isolates were subjected to determination of the minimal inhibitory concentration (MIC) of ENR by use of a commercial gradient strip test (Liofilchem s.r.l., Roseto degli Abruzzi, Italy). *E. coli* ATCC® 25,922™ was used as quality control strain. The applied procedure was in accordance with the manufacturer’s instructions [46]. A 0.5 McFarland turbidity suspension was obtained, measured by optical density, by adding two or three well-separated colonies from a single isolate to a glass tube containing 3.0 mL of sterile PBS. A homogenous bacterial lawn was applied on commercially available Mueller-Hinton (MH) agar plates (Thermo Fisher Scientific, Breda, The Netherlands), by spreading the suspension with a sterile cotton swab (± 100 μL). Finally, the gradient strip tests were placed in the center of the plate. All plates were incubated aerobically at 35 ± 2 °C for at least 18 h before interpretation of the test strips. The results of the gradient strip tests were evaluated visually, by examining the intersection of growth reduction and the gradient strip. The concentration mark coinciding with this intersection was read as the MIC of ENR for the specific strain.

### Characterization of FQ resistance regions

Mutations in the QRDRs are the primary source of resistance against FQs [47]. These QRDRs relate to specific sites on the bacterial DNA, coding for DNA *gyr*ase and topoisomerase IV [48]. Since amino-acid substitutions in *gyr*A-*par*C occur most frequently, mutations in *gyr*A-*par*C were investigated as resistance markers in this study [49]. ,Additionally, the presence of plasmid-mediated resistance via resistance genes *qnr*S, *qnr*A or *qnr*B was assessed [50] by qualitative screening with gel-electrophoresis after PCR analysis. Randomly selected wild-type and non-wild type isolates, collected from the fecal samples, were subjected to PCR characterization (*n* = 17). The protocols and primers used for PCR have been described previously by Chantziaras et al. [51]. Briefly, a MasterCycler Gradient EPS-S Thermal Cycler (Eppendorf AG, Hamburg, Germany) was used for amplification of the genes. After matching with the *gyr*A [52] and *par*C [53] reference sequences of *E.coli* K12 MG1655 [54], all obtained amplicons were sequenced (Eurofins Genomics GmbH, Ebersberg, Germany) and further investigated for point mutations via BioNumerics 7 software (Applied Maths NV, Sint-Martens-Latem, Belgium) and BioEdit 7 multiple alignment tool (Tom Hall, Ibis Therapeutics, Carslbad, USA).

### Pharmacokinetic analysis

Phoenix® WinNonlin® 6.3 (Pharsight-Certara, Princeton, NJ, USA) was used for the pharmacokinetic (PK) analysis of the data. Non-compartmental (NCA) data analysis was performed and following PK parameters (relevant to the pharmacodynamics properties of ENR) were calculated: area under the 24 h-time curve (AUC_0–24h_), area under the 58 h-time-curve (AUC_0–58h_), AUC_0–58h_ normalized for dose (AUC_0–58h_/D), maximal plasma or fecal concentrations of ENR (C_max_), time of maximal concentration (T_max_), steady state plasma or fecal concentrations of ENR (C_ss_). The AUC values were determined using the linear up-log down trapezoidal method.

### PK/PD analysis

The collected data from the in vitro tests was linked to the in vivo PK data (AUC per dosing interval 0–24 h, C_max_) with following calculated PK/PD parameters; C_max_/MIC and AUC_0–24h_/MIC (h).

### Statistical analysis

Plasma, intestinal and fecal ENR concentrations of the four different groups were compared on the different time points using a single-factor analysis of variance (ANOVA) with SPSS 25.0 (IBM, Chicago, IL, USA). Transformation of data (logarithmic or square root) was occasionally applied to achieve the normality assumption. A post-hoc Tukey test was performed to assess the differences between each of the four treatment groups (significance level *p* < 0.05). When equal variances were not assumed, a post-hoc Games-Howell test was performed (significance level *p* < 0.05). No animals were excluded from data analysis.

## Supplementary information

**Additional file 1.**

## Data Availability

The datasets used and/or analysed during the current study are available from the corresponding author on reasonable request.

## Abbreviations

ACN: Acetonitrile
AMR: Antimicrobial resistance
ANOVA: Analysis of variance
AUC: Area Under the Curve
BW: Bodyweight
CFU: Colony Forming Units
CIP: Ciprofloxacin
D: Administered dose
DNA: Deoxyribonucleic acid
ECOFF: Epidemiological cut-off
ENR: Enrofloxacin
ESI: Electrospray ionization
FAMHP: Federal Agency for Medicines and Health Products
FQ: Fluoroquinolones
GIT: Gastro-intestinal tract
H2O: Water
IM: Intramuscular
IS: Internal standard
LC-MS: Liquid Chromatography-Mass Spectrometry
MALDI-TOF: Matrix-Assisted Laser Desorption Time Of Flight
MeOH: Methanol
MIC: Minimal Inhibitory Concentration
MH: Mueller-Hinton
PBS: Phosphate Buffered Saline
PD: Pharmacodynamic
PFGE: Pulsed-field gel electrophoresis
PK: Pharmacokinetic
PO: Oral
QRDR: Quinolone Resistance-Determining Regions
REP-PCR: Repetitive element sequence-based polymerase chain reaction
VICH: Veterinary International Conference of Harmonization
WT: Wild-type
