# Supplementary material for: The impact of therapeutic-dose induced intestinal enrofloxacin concentrations in healthy pigs on fecal Escherichia coli populations
Source: BMC Vet Res. 2020 Oct 8;16:382. doi: 10.1186/s12917-020-02608-9 (PMC7545837; doi:10.1186/s12917-020-02608-9)
Supplement: Supplementary file 1 — Additional file 1. [file 12917_2020_2608_MOESM1_ESM.docx]

**Supplementary files**

**The impact of therapeutic-dose induced intestinal enrofloxacin concentrations in healthy pigs on fecal *Escherichia coli* populations**

Joren De Smet^1^, Filip Boyen^2^, Siska Croubels^1^, Geertrui Rasschaert^3^, Freddy Haesebrouck^2^, Robin Temmerman^1^, Patrick De Backer^1^, Mathias Devreese^1^*

^1^Department of Pharmacology, Toxicology and Biochemistry, Faculty of Veterinary Medicine, Ghent University, Salisburylaan 133, 9820 Merelbeke, Belgium

^2^Department of Pathology, Bacteriology and Avian Diseases, Faculty of Veterinary Medicine, Ghent University, Salisburylaan 133, 9820 Merelbeke, Belgium

^3^Technology and Food Science Unit, Flanders Research Institute for Agriculture, Fisheries and Food, Brusselsesteenweg 370, 9090 Melle, Belgium

* Corresponding Author: Mathias Devreese e-mail: Mathias.Devreese@UGent.be tel: 32 (0)92647347

***Experimental design***


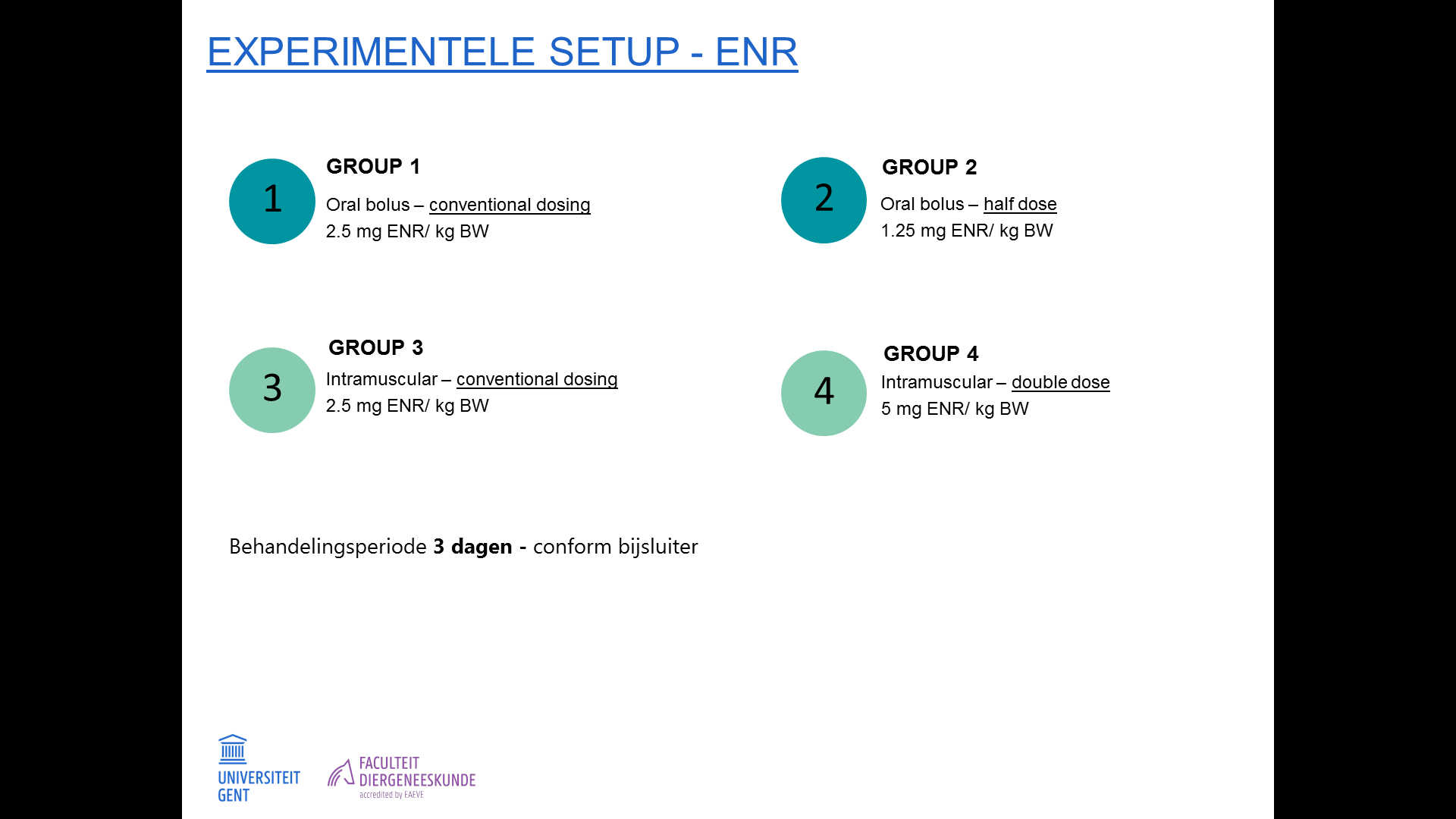


Figure A: Flow-chart of the experimental setup for the enrofloxacin (ENR) administrations with the dose per kg of bodyweight (BW).

***Chromatographic separation and instrumentational parameters***

| Table A1. Gradient elution scheme for chromatographic separation with mobile phases: 0.1% acetic acid in H_2_O (A) and acetonitrile (ACN) (B). Liquid chromatography (LC) was performed on a Surveyer ThermoFisher Scientific system (Breda, The Netherlands). Flow rate was 400 µL/mL. | |
| --- | --- |
| **Time (min)** | **Gradient** |
| 0 – 4 | 85% A, 15%B |
| 4 – 4.5 | Linear to 10% A, 90%B |
| 4.5 – 7 | 10% A, 90% B |
| 7 – 7.5 | Linear to 85% A, 15% B |
| 7.5 – 10 | 85% A, 15% B |
|  | |

| Table A2. Liquid chromatography (LC) and mass spectrometry (MS) instrumentational parameters obtained after direct infusion of enrofloxacin (ENR) and internal standard (IS) ENR-d5 working solutions of 0.01 µg/mL in an H_2_O/acetonitrile (ACN) mixture (80/20 v/v). | |
| --- | --- |
| **LC or MS parameter** | **Optimized value** |
| Column oven temperature | 45°C |
| Autosampler tray temperature | 5°C |
| Spray voltage | 3,800 V |
| Vaporizer temperature | 300°C |
| Sheath gas pressure | 33 au (arbitrary units) |
| Ion sweep gas pressure | 2.0 au |
| Auxiliary gas pressure | 15 au |
| Capillary temperature | 300°C |
| Collision pressure | 1.5 mTorr |
|  | |

***Validation parameters LC-MS/MS method***

| Table B1. Results of major validation parameters: linearity (with correlation coefficient r and goodness-of-fit g), limit of quantification (LOQ, n=6, mean value given ± standard deviation (SD)), limit of detection (LOD, n=6, mean value given ± SD) and signal suppression or enhancement (SSE, n=6, mean value given ± SD) for enrofloxacin (ENR) with calibrator range in feces: 0.050 – 25 µg/g and plasma: 0.035 – 25 µg/mL. Experimental samples always fitted within range. | | | | | |
| --- | --- | --- | --- | --- | --- |
| **Matrix** | r | g | LOQ (µg/g or µg/mL) | LOD (µg/g or µg/mL) | SSE (%) |
| Feces | 0.9983 | 5.07 | 0.050 ± 0.00096 | 0.0057 | 52.65 ± 4.61 |
| Plasma | 0.9978 | 5.59 | 0.035 ± 0.0027 | 0.0020 | 66.25 ± 5.83 |
|  | | | | | |

| Table B2. Validation results for the parameters accuracy and precision based on different theoretical concentration levels. | | | | |
| --- | --- | --- | --- | --- |
| **Matrix** | Theoretical concentration  (µg/mL or µg/g) | Mean concentration ± SD  (µg/mL of µg/g) | Precision  RSD (%) | Accuracy  (%) |
| **Faeces** | 0.050^a^ | 0.0508 ± 0.000964 | 1.9 | 1.7 |
|  | 0.250^a^ | 0.251 ± 0.00537 | 2.1 | 0.4 |
|  | 25.00^a^ | 25.142 ± 1.0778 | 4.3 | 0.6 |
|  | 0.250^b^ | 0.249 ± 0.00529 | 2.1 | -0.4 |
|  | 25.00^b^ | 25.474 ± 1.163 | 4.6 | 1.9 |
| **Plasma** | 0.035^a^ | 0.0363 ± 0.00271 | 7.5 | 3.7 |
|  | 0.250^a^ | 0.251 ± 0.00545 | 2.2 | 0.2 |
|  | 25.00^a^ | 25.910 ± 1.228 | 4.7 | 3.6 |
|  | 0.250^b^ | 0.243 ± 0.0135 | 5.5 | -2.6 |
|  | 25.00^b^ | 25.925 ± 0.690 | 2.7 | 3.7 |
| ^a^ Within-run accuracy and precision (n=6)  ^b^ Between-run accuracy and precision (n=6)  SD: standard deviation; RSD: relative standard deviation. Acceptance criteria: *accuracy*: > 0.01 µg/mL or µg/g: −20% to +10%; *within-run precision*: *RSD_max_*: ≥ 0.01 µg/mL or µg/g and < 0.1 µg/g or µg/mL: 15%, ≥ 0.1 µg/mL or µg/g: 10%; *between-run precision*: RSD_max_ = 2^(1-0.5logC)^ (with C (10-9) the concentration at which the samples were fortified). | | | | |
